# Attitudes and access of Irish general surgery trainees to robotic surgical training^☆^

**DOI:** 10.1016/j.sopen.2022.03.010

**Published:** 2022-03-30

**Authors:** Lauren V. O'Connell, Cathal Hayes, Mohamed Ismail, Diarmuid S. O'Ríordáin, Adnan Hafeez

**Affiliations:** Department of Surgery, Beacon Hospital, Sandyford, Ireland

## Abstract

**Background:**

Although the use of robotic-assisted surgery is now mainstream for procedures such as robotic prostatectomy and hysterectomy, its role in general surgery is less well established. Access to training in robotics for general surgery trainees in the Republic of Ireland is variable. Further, there are no data on specific attitudes of Irish trainees toward the role of robotics. We aimed to establish attitudes of Irish general surgery trainees toward the perceived utility of robotic surgery as well as access and satisfaction with training.

**Methods:**

A survey was disseminated to trainees in the Republic of Ireland enrolled in a General Surgery training scheme via email and social media. Data collected included stage of training, intended subspecialty, interest in developing robotic skills, previous exposure to robotic surgery, satisfaction with current access to robotic training, and opinion on formally incorporating training in robotics into the general surgery curriculum.

**Results:**

The response rate was 53.8%. Of these, 83% reported interest in training in robotics and 66% anticipated using the technology regularly in consultant practice. Previous exposure to robotic-assisted surgery was significantly predictive of interest in developing the skillset (P = .014). More than 71% of trainees reported that they were not satisfied with access to robotic training. Of those satisfied with access, 40% felt there was a role for incorporating robotic training into the curriculum compared to 68% of those dissatisfied.

**Conclusion:**

Irish general surgery trainees perceive robotic-assisted surgery to be highly relevant to their future practice. There is an unmet need to provide additional training in the skillset.

## Highlights

• Irish general surgery trainees perceive robotic surgery (RS) to be highly relevant to their future practice.
• Access to training in RS is limited, and the majority of trainees desire more access to RS training.
• Most trainees feel that there is a role for incorporating RS training into the curriculum.

## INTRODUCTION

The benefits of a minimally invasive surgical approach over open are well enumerated, including minimisation of intraoperative blood loss, reduced postoperative pain, and a shorter length of stay [1]. Technical innovation in surgery has facilitated the emergence of robotic surgery (RS) as a tool to enhance the performance of minimally invasive surgery. Key benefits of the technology include a stable platform, 3-dimensional vision, and articulated instrument wrists permitting precise dissection in confined anatomical spaces. Although adoption of RS by subspecialties such as urology and gynaecology has been rapid and successful, with robotic prostatectomy and hysterectomy now standard procedures, uptake in general surgery has been slower. Nevertheless, utilisation of RS in general surgery is rapidly expanding, with a 10–40-fold increase in its use relative to laparoscopy in 2017 [2].

The emergence of a novel surgical technique brings with it attendant difficulties with respect to training. It emerges in the context of preexisting well-defined issues with surgical training such as working hour duty restrictions and diminishing trainee operative volume and case complexity [[3], [4], [5]]. Implementation of robotic surgery training carries with it associated issues of financial cost, the need to balance training and increased operative case time with appropriate turnover of resources such as theatre space, and maintenance of satisfactory patient outcomes [6]. Because of the relative recency of the technology, senior surgeons themselves may be on a learning curve with RS and consequently more cautious to entrust trainees to directly perform procedures. Finally, not all institutions will have access to RS.

The factors outlined above are also influenced by geographical and regional variables. Although literature exists on the attitudes of general surgery trainees toward RS, the majority of these data are produced from North America [7,8]. The majority of surgical Da Vinci robotic systems in use worldwide are in the United States (66%), with less than one third of this number (17%) in use in Europe [9]. Consequently, access to RS in this region for trainees is greater, with 98% of colorectal (CRS) residency programs in the United States and Canada indicating that they utilised RS [10]. In addition, 74% of CRS programs in United States and Canada had a formal RS training curriculum for CRS residents, the majority based on the Society of Gastrointestinal and Endoscopic Surgeons 2006 consensus document outlining the desirable components of such a curriculum [10]. In contrast, the Intercollegiate Surgical Curriculum Programme for surgical trainees in the United Kingdom and Republic of Ireland at present does not formally incorporate aspects of robotic training as distinct from minimally invasive procedures.

To date, no specific data exist on attitudes of General Surgery trainees in the Republic of Ireland toward the utility of robotic surgery and their satisfaction with access to training in RS. It is also currently unclear whether trainees in the region feel formal incorporation of RS into the curriculum would be of utility or superfluous.

We therefore aimed to investigate the attitudes of Irish General Surgery trainees toward the above.

## METHODS AND MATERIALS

Ethical approval was sought and obtained from the Beacon Hospital Research Ethics Committee prior to commencing the study. An online 15-point survey was designed using a commercially available web-based platform. This was reviewed by the Training Programme Director for General Surgery and subsequently forwarded by email from the training body responsible for supervising postgraduate surgical training (Royal College of Surgeons in Ireland) to all Higher Surgical trainees (ST3–5) enrolled in a General Surgery training scheme, including those who were out-of-program for research. This encompassed trainees with a subspecialty interest in Colorectal, Upper GI, Bariatric, Breast-Endocrine, Hepatobiliary, and Trauma surgery. Core surgical trainees (ST1–2) and surgical trainees in HST training programs other than General Surgery were not included. The survey was also disseminated by social media (WhatsApp and Twitter).

Respondents were advised that all responses were anonymous and that individual responses would not be shared. No incentive was offered in reward for survey completion.

Data collected included age, sex, stage of training, and intended subspecialty interest. Respondents were also asked to report on their prior exposure to robotic surgery; access to robotic surgery in their current institution, including simulation training; access to robotic surgery by subspecialty; and their interest in pursuing robotic training. They were also asked whether they anticipated utilising robotic-assisted surgery regularly in their future consultant practice, their satisfaction with their current access to robotic training in their institution, and their opinion on the value of formally incorporating robotic surgery training into the general surgery curriculum (see Table 1 for full list of survey responses). Other than demographic data, responses were either binary or on a Likert scale.

**Table 1:** List of survey questions and responses

*Number*	*Question*	*Response options*
1	Age	26–30; 31–35; 36–40; other
2	Sex	Male/Female
3	Stage of training	ST 3–8, fellow, out-of-program for research
4	Subspecialty interest	Upper GI/bariatric, colorectal, hepatobiliary, breast/endocrine, trauma
5	Do you anticipate using robotic surgery regularly in your future consultant practice?	Yes/No
6	Are you interested in improving your robotic skills/acquiring further training in robotic surgery?	Yes/No
7	Do you think training in robotic-assisted surgery is relevant to you as a general surgery trainee?	Yes/No
8	Is there robotic-assisted surgery in the institution where you are currently working	Yes, including for general surgery cases Yes, but not used for general surgery No
9	Have you observed but not scrubbed for robotic surgery cases?	Yes/No
10	Have you scrubbed in for robotic cases as an assistant?	Yes/No
11	Have you performed a robotic-assisted case (or part of a case) under supervision?	Yes/No
12	Have you had access to robotic simulation training?	Yes/No
13	Have you attended any courses on robotic-assisted surgery?	Yes/No
14	Are you satisfied with your current access to robotic training?	Yes/No
15	Do you feel there is a role for formally incorporating robotic surgery training into the general surgery curriculum?	Yes Not currently, but possibly in the future No

Responses were collected over a 4-month period from December 2020 to March 2021. A reminder was sent by email and via social media at 6 weeks post the date of initial circulation of the survey. All responses were collected and managed via a secure web-based platform. To avoid potential identification of individual responses, all data was analyzed after closure of the survey. Respondents were not asked to identify their current or previous institutions.

Chi-square or Fisher exact test, as appropriate, was used to evaluate related differences between categorical data. An unpaired *t* test was used to evaluate Likert scale data responses. Statistical analysis was performed using GraphPad Prism v.9.1.0 (San Diego, CA, USA). All tests were 2-sided.

## RESULTS

The overall response rate was 53.8%, with 35 responses received in total (*n* = 35). Of the 35 responses received, all completed the survey in full. This comprised 26 (74%) men and 9 women (26%). Thirty-four percent of respondents were in the 26–30 and 31–35 age categories, respectively, with a further 31% in the 36–40 category.

The majority of respondents, 34%, were in the first 2 years of specialty training (ST3–4), with 23% midway through (ST5–6) and 29% in the final years of training (ST7–8). The remainder were either on time out of specialty training to complete formal postgraduate research or on fellowship.

Colorectal surgery was the most commonly declared subspecialty interest (46%) followed by Upper GI/Bariatric (31%), Breast/Endocrine (11%), Trauma (6%), and Hepatobiliary surgery (6%).

With respect to access to RS, 34% of respondents stated that there was no robotic surgery performed in their current institution, whereas a further 22% reported that although RS was performed, it was not utilized for general surgery operative cases. Nevertheless, 42% of respondents currently worked in an institution in which there was access to RS for general surgery cases.

When queried about their previous exposure to and extent of robotic training, the majority of trainees affirmed that they had observed (60%) or scrubbed in for (57%) at least 1 robotic-assisted operative case. However, only 14% had performed a case or were part of a case at the console. Forty-six percent had had access to simulation training, whereas 23% had attended a course on robotic-assisted surgery. All of those trainees who had performed a robotic-assisted case had had access to simulation training. Trainees who worked in an institution where the robotic platform was utilized by General Surgery trainers were significantly more likely to indicate exposure to RS in the form of either observing (*P* = .0069) or assisting (*P* = .0369) in cases. However, no difference was detected for performance of cases (*P* = .365).

With respect to interest in training in RS, 83% indicated interest in training in RS, whereas 66% anticipated using the technology regularly in their future practice as a consultant. There was no significant difference in interest in robotic training by either intended subspecialty (*P* = .36) or program stage (*P* = .59).

Previous exposure to RS (assisting, simulation training, or performing, but not observation alone) was significantly predictive of interest in developing the skillset (*P* = .014) and of trainee expectation that they would utilize robotic surgery regularly in consultant practice (*P* = .024).

More than 71% of trainees (*n* = 25) reported that they were not satisfied with their current access to robotic training. Of those satisfied with access, 40% felt that there was a role for incorporating robotic training into the curriculum compared to 68% of those dissatisfied. However, this discrepancy was not statistically significant (*P* = .134). In total, 60% percent of trainees surveyed wished formal training in RS to be incorporated into training. Another 28% indicated that although they felt that this was not currently indicated, it may become so in the future. Only 11% of trainees stated definitively that they perceived no need for formalised RS training.

## DISCUSSION

This study reports on the attitudes of General Surgery trainees in the Republic of Ireland toward the utility of robotic surgery in their current and future practice as well as their access to training in this modality. This was a national study with a 54% response rate.

To date, most work on the attitude of surgical trainees to robotic surgery is derived from North America, whereas there are scanty data in the literature currently examining the European and UK experience. This likely reflects the differential uptake of robotic surgery in these regions; in 2015, a total of 520 robotic surgical systems were in use throughout Europe, including 45 located in the United Kingdom, compared to 2,100 in the United States. None were in use in Ireland in university or training hospitals at that time [11]. In addition, the majority of reports evaluating access to training in robotics for residents address the needs of urology or gynaecology trainees, reflecting the earlier adoption into practice and more established role of RS in these subspecialties [[12], [13], [14], [15]].

In total, there are nine robotic platforms across public hospitals in Ireland, all in Model 4 hospitals. Of these, three are dual-console platforms which afford greatly enhanced training opportunities, with a fourth dual-console platform awaiting delivery [16,17]. A further five platforms are available in the private sector. Our data demonstrate that the majority of Irish trainees have had some exposure to RS during their training, either observing a case (60%) or as bedside assistant (57%). However, less than a quarter of these (14%) had ever had the opportunity to be supervised during a case at the console. As anticipated, trainees who reported that they worked in an institution in which a robotic platform was utilised for general surgery had significantly higher exposure in the form of either observing or assisting for a robotic case. However, there was no difference in the rates of those who had performed a case, suggesting potential barriers to proctoring trainees beyond simple lack of access to the platform. The participation rate of console operating for Irish trainees is thus far lower than for their North American peers. In comparison, 2019 data reported that 92% of US training programs had active participation of general surgery residents in RS-assisted cases, whereas a similar 2020 survey of US residency programs reported 95% of these with active participation in RS by colorectal trainees [10,18]. Access to an institution where RS was performed was also limited, with less than half of trainees holding posts in a unit where RS was utilised for general surgery cases. Again, this compares unfavourably with current US data, where 98% of institutions had a robot in use and 78% reported having at least one dual-console robotic system to facilitate robotic surgical training [10]. Training opportunities in RS are greatly facilitated by dual-console platforms, which permit the addition of visual cues onscreen, rapid switching of the operator, and proctoring of cases, which are not feasible with single-console platforms. Although the majority of robotic platforms in Irish hospitals are single-platform, the proportion of dual-console platforms is increasing over time, with only one such platform in use five years ago [16,17].

The perceived relevance of RS to trainees has undergone a substantial shift over recent years as uptake has increased. Data from a 2013 survey of US general surgery residents revealed that 40% were unsure if they would use robotic-assisted surgery in their consultant practice, whereas close to 25% felt that they definitely would not. Despite this, a clear majority of residents (81%) expressed interest in receiving formal RS training [8]. A similar survey 5 years later demonstrated 97% of residents expressing interest in RS training and 77% anticipating use in consultant practice [7]. Our data are in line with this most recent evaluation, with a definite majority of trainees (66%) anticipating that they would use RS regularly in consultant practice and 83% indicating interest in training.

The limited access to RS training for Irish trainees described herein and supported by recent data from trainee representative bodies [19] is concerning, particularly in the context of the rapid expansion in use of the technology. Our data are concordant with the existing body of literature which shows that this shift in practice is clearly appreciated by a majority of trainees, who perceive the technology to be highly relevant to their future practice and express a desire to receive training in the modality [7,8,10]. Although the evidence base for RS in general surgery is as yet by no means definitive on its benefits, use of the tool is only likely to increase as more platforms become available, accessibility improves, and the associated costs are reduced [20,21]. Arguably, the optimal time period in which to acquire proficiency in robotic skills is during surgical training to mitigate the attendant adverse event risks presented by entering consultant practice while still in the early phase of a learning curve [22,23].

Finally, a majority of respondents (60%) felt that there was a role for incorporating robotic training into the general surgery curriculum. No significant difference in this response was noted between those satisfied with their current access to RS training and those dissatisfied. However, a trend was noted favouring incorporation into the curriculum by those not satisfied with access to RS training. Despite the increasingly widespread use of RS over the past years and an updating of the Intercollegiate Surgical Curriculum Programme as recently as August 2021, there is no acknowledgement of the role of RS or a requirement to engage in robotic training. By contrast, laparoscopic surgery trainees are mandated to achieve a variable degree of proficiency for named index procedures, dependent on their subspecialty interest [24].

In comparison, in 2019, 68% of general surgery residency programs in the United States had a formal robotic surgery component in their training curriculum, although the content of these varies substantially [18,25]. Outside of North America, fellowship programs in Australia in centers experienced with RS have also successfully introduced a defined robotic training curriculum with progression points and objective standardisation [22]. In Europe, similar defined RS curricula exist for trainees in urology [13,14]. Given the likelihood and expectation that many of today's general surgery trainees will engage in RS in their future consultant practice, delivery of formalised structured training with stepwise advancement based on objective competency acquisition at a trainee level is highly desirable to ensure safe progression to independent practice.

This paper does have several limitations which are typical of survey data. These include a small sample size and the potential for response bias. Trainees with a prior interest in RS may be more likely to respond, skewing responses toward those positively inclined toward the utility of the platform. Although the absolute number of respondents is small, the total number of General Surgery trainees in higher specialist surgical training at any one time in the Republic of Ireland is also small (approximately 60–70), and the response rate is in line with previous studies published on this topic. There was a reasonably even distribution of respondents by stage of training and subspecialty interest. Although the views of more junior surgical trainees (ST1–2) are not represented in these data, we felt that exposure to RS among this group was likely to be low and that anticipated future use or relevance of the platform may be more difficult to assess prior to selection of subspecialty.

In conclusion, Irish general surgery trainees perceive robotic surgery to be relevant to their training and anticipate that it is likely to form part of their future consultant practice. The majority of trainees feel that their current access to robotic training is inadequate. As the current surgical curriculum does not specifically incorporate robotic surgical training, there is an unmet need to provide additional dedicated training in the skillset.

## Author Contributions

All authors contributed to the manuscript in conceptualisation, data collection and analysis, supervision, or editing.

## Conflict of Interest

The authors declare no conflict of interest.

## Funding Source

None declared.

## Ethical Statement

Appropriate ethical approval was sought and obtained prior to commencing the study from the Beacon Hospital Research Ethics Committee.
